# Heavy Metal Mixture Exposure and Effects in Developing Nations: An Update

**DOI:** 10.3390/toxics6040065

**Published:** 2018-11-02

**Authors:** Brilliance Onyinyechi Anyanwu, Anthonet Ndidiamaka Ezejiofor, Zelinjo Nkeiruka Igweze, Orish Ebere Orisakwe

**Affiliations:** 1 World Bank Africa Centre of Excellence in Oilfield Chemicals Research, University of Port Harcourt, PMB, 5323 Port Harcourt, Rivers State, Nigeria; brillianceanyanwu@gmail.com; 2 Department of Experimental Pharmacology & Toxicology, Faculty of Pharmacy, University of Port Harcourt, PMB, 5323 Port Harcourt, Rivers State, Nigeria; ndidiezejiofor@yahoo.com; 3 Department of Experimental Pharmacology & Toxicology, Faculty of Pharmacy, Madonna University Elele, PMB, 5001 Elele, Rivers State, Nigeria; zeligweze@gmail.com

**Keywords:** heavy metal mixtures, environmental pollution, public health effects, Sub-Saharan Africa, epidemiology

## Abstract

The drive for development and modernization has come at great cost. Various human activities in developed and developing countries, particularly in sub-Saharan Africa (SSA) have given rise to environmental safety concerns. Increased artisanal mining activities, illegal refining, use of leaded petrol, airborne dust, arbitrary discarding and burning of toxic waste, absorption of production industries in inhabited areas, inadequate environmental legislation, and weak implementation of policies, have given rise to the incomparable contamination and pollution associated with heavy metals in recent decades. This review evaluates the public health effects of heavy metals and their mixtures in SSA. This shows the extent and size of the problem posed by exposure to heavy metal mixtures in regard to public health.

## 1. Introduction

Pollution in the environment is the price we have paid for growth in industrialization and urbanization. While advancement in technology has improved the standard of living, it has also released unwanted substances into the environment, thereby raising issues with public health. Ineffective regulations on pollution and emission controls due to increasing urbanization and industrialization have put humans at risk. Sub-Saharan Africa (SSA) has become heavily polluted with heavy metals and other chemicals [1].

Heavy metals are persistent environmental pollutants and humans are exposed to them through water, air, food, or industrial settings [2]. Natural and anthropogenic activities are the two sources of heavy metal pollution. Biological buildup in the food chain allows multi-heavy metal pollutants to increase [3]. Heavy metals are extensively used to uphold the standard of living in developed nations and they enter the environment through natural and anthropogenic sources, including artisanal mining, illegal refining, inadequate disposal of waste, and the constant increase in industrialization and urbanization. Thus, the risk of human exposure continues to increase as a result of the prevalence of heavy metals in the environment. Insufficient control of reclaim plans has led to unplanned exposure in the past [2]. Metal poisoning from various sources is a significant problem, from evolutionary, natural, and dietary perspectives [4,5].

The ubiquity of heavy metals poses major public health threats to adults and children. While toxicity from industrial exposure usually affects several organ systems, the severity of the health outcomes is dependent on the nature of the metal, the method of exposure, the age of the individual, and finally, the person’s individual susceptibility [6]. Humans are exposed to heavy metals, either voluntarily or involuntarily, from various sources resulting from an increase in industrial pollution, manmade or natural activities. According to the authors of Martinez-Finley et al. [7], there is a constant increase in heavy metal contamination around the globe and this has posed serious health concerns. Metals exist as mixtures in the environment [8]. Given that Pb, Hg and Cd are widely distributed in the environment through various sources, human exposure is inevitable because these metals are non-degradable, environmentally persistent, and can accumulate in ecosystems at very low levels.

The increase in population, urbanization, and industrialization, coupled with the rapid growth of buildings as a result of inadequate planning, have caused an increase in the production of waste without proper disposal systems [1]. Increasing artisanal mining activities, illegal refining, use of leaded petrol, airborne dust, arbitrary dumping, and burning of toxic waste, absorption of production industries in inhabited areas, as well as weak and insufficient environmental legislation, have all given rise to the unparalleled heavy metal pollution in past years [9]. According to UNEP [10], which is a report on the environmental assessment of Ogoniland, Rivers State, Nigeria, the levels of hydrocarbon and heavy metals in the soil, drinking water, and air in 10 communities are almost 1000 times higher than the permissible levels.

There were higher levels than the World Health Organization WHO recommended limits for blood Pb [11] Ni, Cr, and Mn [12] in pregnant women and children in Nigeria. Similarly, the concentration of heavy metal pollution in South Africa in maternal and umbilical cord blood from inhabitants of preferred areas showed intolerably high levels of Hg, Pb, Cd, and Se [13]. Heavy metals were found in the umbilical cord whole blood samples indicating high risks of heavy metal pollution to both adults and fetuses. Given the prevalent heavy metal exposure from both environmental and occupational settings in developing countries, an intimate knowledge of the effect of these noxious metals on public health is important for drafting robust policies for preventative medicine in Africa.

The present review provides an overview of the mechanisms of organ toxicity and public health effects of heavy metal mixtures arising from both occupational and environmental exposures in developing countries and SSA.

## 2. Materials and Methods

### 2.1. Database Searching and Search Strategy

To identify the papers focusing on heavy metal mixture exposure and public health effects in developing nations, we systematically reviewed Google scholar, Research Gate, Springer, Medline and PubMed databases by using the following key search words: ‘heavy metal mixtures’, ‘heavy metal risk assessment’, ‘heavy metal pollution in developing countries’, and ‘heavy metal public health effects’. The search was done independently in each database and the literature was pooled together afterwards. The collected research was scrutinized and double citations were excluded. Results were collated and studied by extracting relevant information.

### 2.2. Inclusion and Exclusion Criteria

The addition and omission yardstick was adopted when evaluating the title and abstract of each journal article. Articles were included if they detailed a relationship between heavy metal mixture exposure and public health effects, or heavy metal pollution in SSA. Additionally, mechanisms of action of these metal mixtures and their risk assessment in the environment were included. If two or more reports were published from the same study, then only the most recent study or the study with the best assessment of the metal mixture was included. Articles that detailed epidemiological studies were added to the review. Studies reporting metals as being beneficial to the body were excluded from the study. Additionally, the review excluded articles that were not written in the English language.

**Figure 1:**
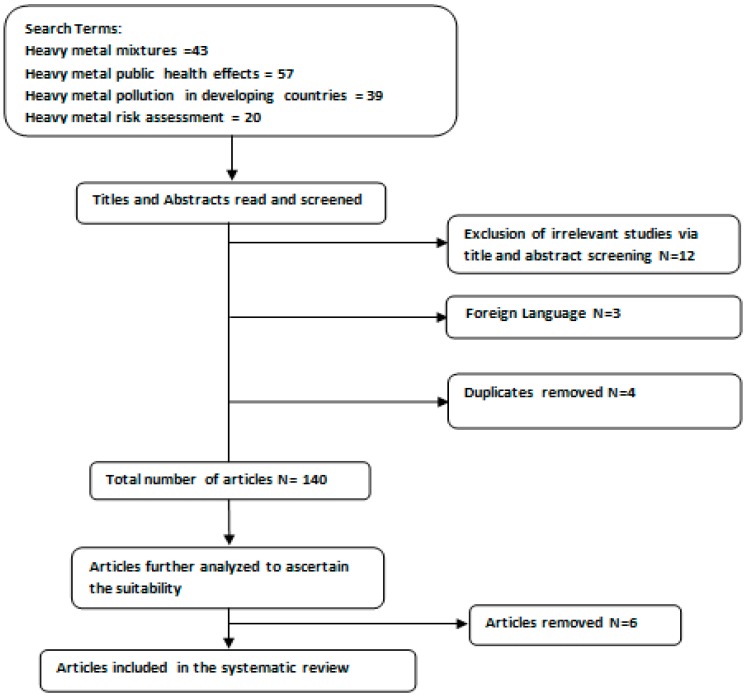
Study selection flow diagram.

Initially, a total of 159 articles were kept for this review. After collecting the results and removing 4 duplicates, a total of 155 articles were kept for data retrieval. In the course of extracting relevant data for the study, 12 articles were omitted as they were not applicable to the focus of this review; three articles were removed because they were written in foreign languages; leaving a total of 140 articles for this review. In order to ascertain the suitability of the articles, six more articles were removed, as shown in Figure 1 below. After applying the selection criteria, 134 articles were retained and used in this systematic review. These articles explored the association between low dose metal mixture exposure and health effects in developing countries.

## 3. Exposure to Heavy Metal Mixture

Cd, Hg, Pb, Cr, and Ni are all toxic metals that are persistent in our environment [14] and they cause toxicity in different environmental media such as the soil, plants, air, wildlife, water, and domestic animals. In toxicology, the method of uptake of metals is through ingestion, inhalation, and skin contact. The absorption by an organism occurs either by diffusion or through conveyers [3]. The health risks posed by these metals are determined by several factors including age, gender, individual susceptibility, route of exposure, and duration of exposure.

**Figure 2:**
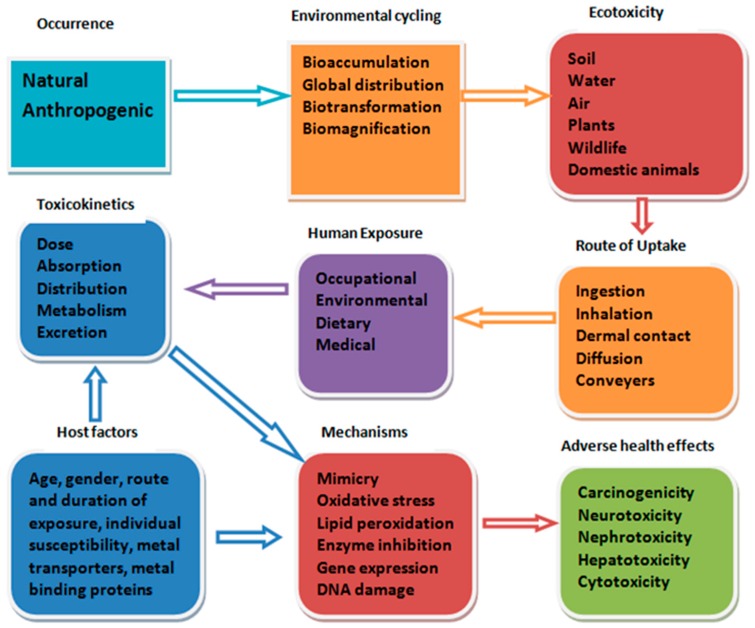
Conceptual framework of human exposure to heavy metal mixture from the environment.

In different environmental media, the joint toxicity of metals is linked with several processes caused by the interaction of the toxicant outside the organism, which introduces the issue of metal speciation, binding, and transport of toxicants. Processes such as absorption, distribution, metabolism, and excretion take part in the uptake and removal of metals. According to Lokke et al. [15], estimating the possible interaction that may occur improves on what is known about the kind of mechanism involved in the toxicity of mixtures. Figure 2 illustrates how humans become exposed to heavy metals from both natural and manmade activities and the possible adverse health effects that may arise through various mechanisms.

## 4. Effects of Heavy Metal Mixture to Tissues and Organs

### 4.1. Cytotoxicity

Healthy living cells can either be provoked to undergo accidental cell death (necrosis) or programmed cell death (apoptosis) by using a cytotoxic compound including heavy metals. Cytotoxicity becomes the capacity of some chemicals or mediator cells to destroy living cells. The co-exposure of Pb and Cd to secluded red blood cells of the common buzzard (Buteo buteo) showed that apoptosis is the main type of cell death resulting from this exposure [16]. A study by Jadhav et al. [17] examined the genotoxic effects of subchronic exposure through drinking water to a mixture of eight metals (Pb, Hg, Cd, Cr, Mn, As, Fe, and Ni) and found that exposure to this mixture produced genotoxicity in rat bone marrow and spleen cells in relatively high doses (10× and 100×). The results also showed that the cytogenetic effects were associated with a dose-dependent increase in lipid peroxidation (LPO) and decrease in the enzymatic and non-enzymatic antioxidative systems in the spleen. Cytogenetic effects induced by the mixture in bone marrow cells indicate its toxic consequences on bone marrow. The mixture components were reported to produce cytogenetic damage, including chromosomal aberrations, micronuclei induction, and sister chromatid exchange (SCE) in bone marrow and other cells [17]. Their study, therefore, concluded that the observed cytogenetic effects of the metal mixture might relate to oxidative stress-induced damage to DNA, interference with the DNA repair process and substitution of cellular essential metal ions [17].

When the body is sick, it is typified by behavioral, autonomic and endocrine changes that are activated by soluble mediators known as pro-inflammatory cytokines that are produced at the site of infection by activated accessory immune cells [18]. These mediators include interleukin-1alpha and beta (IL-1a and IL-1b), tumor necrosis factor-alpha (TNF-a), and interleukin-6 (IL-6). They are significant in coordinating the local and systemic inflammatory response to microbial pathogens. They also act on the brain to cause behavioral symptoms of sickness [18].

The intensity and duration of sickness behavior are regulated by anti-inflammatory cytokines possibly by inhibiting pro-inflammatory cytokine production and decreasing pro-inflammatory cytokine signaling [19]. Studies have shown that administration of IL-10 or insulin-like growth factor 1 (IGF-1) decreases behavioral signs of sickness induced by centrally injected lipopolysaccharide (LPS) [20,21]. Ageing is typified by an elevated activity of the innate immune system, which at the brain level translates into an improved manufacture of pro-inflammatory cytokines like IL-6, and a reduced production of anti-inflammatory cytokines such as IL-10 [22,23].

### 4.2. Oxidative Stress

According to Fowler et al. [24], oxidative stress may possibly be a key factor of the mechanism of toxicity of the metal mixture. Many definitions have been given to oxidative stress but the more useful one is described as a state where oxidative force becomes higher than the antioxidant systems due to loss of the balance between them [25]. This definition is generally accepted because oxidative stress is actually useful in some cases which include inducing programmed cell death to prepare the birth canal for delivery and also strengthening of biological defense mechanisms during physical exercise and ischemia [26].

**Figure 3:**
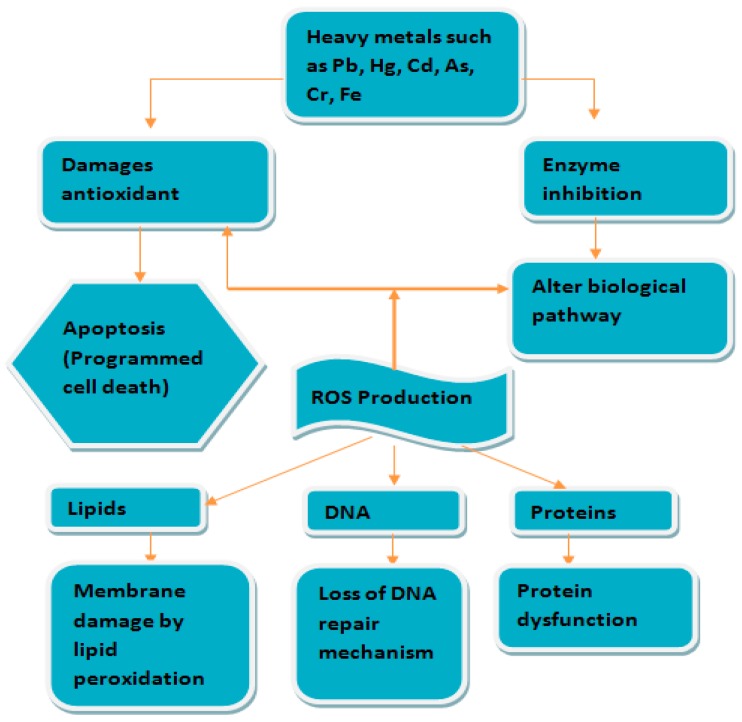
Attack of heavy metals on a cell resulting in the production of reactive oxygen species (ROS).

Jadhav et al. [27] concluded that oxidative stress and lipid peroxidation were induced in several visceral tissues of rats on sub-chronic exposure to a mixture of metals. Several studies have shown that oxidative damage and lipid peroxidation in the liver, brain, and kidney of rats by continuous exposure to Pb [28], As [29], and Cd [30] through water was activated by the production of reactive oxygen species (ROS). Studies have also revealed that mercury, chromium, nickel, iron, and manganese that also induce lipid peroxidation (LPO) and show their toxicity through the generation of reactive oxygen species [31,32,33]. At high concentrations, ROS may cause structural damage to cells, proteins, nucleic acid, membranes, and lipids, leading to a stressed situation at the cellular level [34]. This is illustrated in Figure 3 below.

The production of reactive oxygen species and reactive nitrogen species were suggested as the underlying factor in estimating the toxicity of these metals [35]. Therefore, oxidative damage to tissues through enhanced lipid peroxidation can possibly be the result of a joint effect of the buildup of reactive oxygen species, ensuing from dysfunction GSH and antioxidases, and overproduction of free radicals during sub-chronic exposure to metal mixture [27].

According to Jadhav et al. [27], the inverse association between the levels of GSH and LPO in brain, kidney, and liver suggest that reduction of GSH was a significant cause of LPO. On the contrary, the overproduction of ROS can possibly cause a reduction of GSH. The superoxide dismutase (SOD) can be up-regulated by overproduction of ROS, and its inadequate expression in SOD knock-out results in oxidative stress [36].

Another study by Bhattacharyya et al. [37] revealed a negative correlation of LPO with SOD or GPX or catalase (CAT) in the brain, liver, and kidney and suggested that these antioxidant markers contribute to the oxidative stress-dependent toxicity caused by the metal mixture. The reduction in the activities of antioxidant enzymes may be because of their depletion in response to metal mixture-induced oxidative stress. A study by Jadhav et al. [27] suggested that the oxidative stress induced by subchronic exposure to metal mixture might be linked to the Fenton reaction mechanism and linked with the attenuation in the antioxidative capacity of the rats.

### 4.3. Immunotoxicity

The immune system is a multifaceted system of cells with many vital roles, which are controlled by soluble glycoproteins, the lymphokines [38]. Lymphokines are produced by immunocompetent cells, lymphocytes, and monocytes, but are also secreted by endothelial and epithelial cells [39,40,41]. The immune system functions to protect the body against bacteria, fungi, parasites, and viruses, and also destroy malignant cells or virus-producing cells.

Immunotoxicity is referred to as the deleterious effects of foreign compounds from occupational or environmental exposure characterized by either a suppression or enhancement of the immune system. Observations in epidemiological and experimental studies have shown that a number of environmental and industrial chemicals including heavy metals can adversely affect the immune system [42].

Immunotoxicity is a significant health risk of exposure to heavy metal [27]. As the effect of exposure to individual heavy metals is quite different from the combined effect of the metal mixtures, several studies have demonstrated whether subchronic or chronic exposure to metal mixtures can cause immunotoxicity to humans and experimental animals. A study [27] examined whether subchronic exposure to a mixture of eight metals (Pb, Hg, Cd, Cr, Mn, As, Fe, and Ni) can induce immunotoxicity in male albino rats. Their findings suggested that hematopoietic and immune systems are toxicologically susceptible to the mixture, which could lead to anaemia and suppression of humoural and cell-mediated immune responses in male rats.

Rajeshkumar et al. [43] studied the effects of exposure to a mixture of Cd, Cr, and Pb on biochemical, immunotoxicity level, and morphological features of different tissues of a biomarker freshwater fish at low concentrations and revealed that exposure of aquatic life to this metal mixture (Cd, Cr, and Pb) can change the cytokine alterations leading to immune suppression as well as immune dysfunction. Jung et al. [44] examined a group of metal workers exposed to a variety of metals including Pb, Cd, and Cu in their workplace and concluded that exposure to Pb and Cu in levels below or approximate the current threshold limit value (TLV) lead to clear immunotoxic effects.

### 4.4. Hepatotoxicity

The liver plays a significant role in the metabolism and removal of foreign compounds, which makes it vulnerable to their deleterious effects [45]. Hepatotoxicity refers to damage to the liver resulting from various chemicals and xenobiotics including heavy metals and their metabolites [46]. Symptoms associated with hepatotoxicity include jaundice leading to yellowing of the skin, eyes, and mucous membranes due to high level of bilirubin in the extracellular fluid, severe abdominal pain, generalized itching, nausea, fatigue, weakness, skin rashes, edema, and increase in weight in a short period of time, dark urine and light colored stool [47]. The liver performs more than 500 metabolic functions in the body [48]. The major role played by the liver in the clearance and transformation of chemicals exposes it to harmful effects [49].

Due to the ubiquity of metals in the environment, Bhattacharjee et al. [50] evaluated the effects of long-term exposure at a low dose to a mixture of Cd, As, and Pb and concluded that chronic exposure to a mixture of heavy metals at a very low environmentally relevant dose produced hepatotoxic effects in albino rats. Hepatoxicity has also been evidenced by Yuan et al. [51] to be among the toxicities resulting from the mixture of Pb and Cd on Sprague Dawley rats.

**Figure 4:**
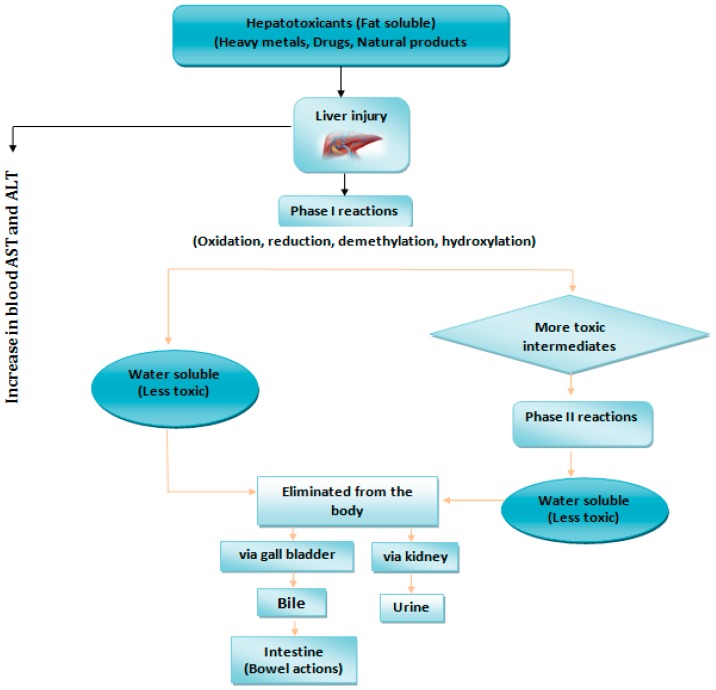
Biotransformation of hepatotoxicants.

The liver could be exposed to high levels of foreign compounds and their intermediates. Metabolism of these foreign compounds can change the properties of hepatotoxicants by either elevating its toxicity (metabolic activation or toxication) or depleting its toxicity (detoxification) [52]. Figure 4 shows the biotransformation of hepatotoxicants.

Reactions associated with phase I produce toxic metabolites which become innocuous by phase II reactions. Phase II reactions entail the linking of chemicals with water-soluble moieties leading to a more water-soluble metabolite [52]. Lee [53] reported glutathione’s capacity to covalently bind to toxic metabolites by glutathione-S-transferase as another phase II reaction. Thus, these reactions are considered as detoxification pathways. Yet, phase II reactions can result in the production of unbalanced precursors to reactive species that can cause hepatotoxicity [54].

An epidemiological study on the joint toxicity of heavy metal mixtures in human liver cells has been conducted by Lin et al. [55]. The study exposed the liver cells to a mixture of eight metals, which included Hg, Cr, Pb, Cd, Cu, Zn, Mn, and nickel, and suggested the need to consider the assessment of the risk of co-exposure heavy metal contamination after the side effects of these metals on the liver cells.

### 4.5. Nephrotoxicity

Today, chronic kidney disease (CKD) has become one of the most prevalent diseases in the world. It is characterized by a permanent loss of nephrons and finally ends with a reduction in glomerular filtration rate (GFR) [56]. It is estimated that eight-16% of the world’s populace is affected by some form of CKD [57]. Metals such as As, Pb, Hg, and Cd are persistent environmental toxicants and are known nephrotoxicants [58]. A decrease in total glomerular filtration rate (GFR) causes foreign compounds and toxicants to accumulate in the blood and result in metabolic distortion and/or organ intoxication [59].

**Figure 5:**
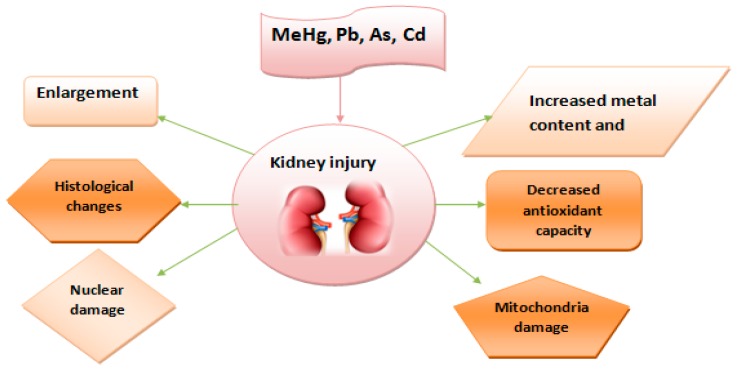
A conceptual framework of metal mixture effects on the kidney.

Owing to the ubiquity of heavy metals in the environment, individuals are exposed continuously overtime to pollutants that have the capacity to negatively affect various organs in the body system including the kidney leading to enlargement, histological changes, nuclear damage, mitochondria damage, decreased antioxidant capacity, and increased metal content and malondialdehyde (MDA). This is shown in Figure 5 below.

Yuan et al. [51] established that damage to the kidney was seen when rats were exposed to a mixture of Pb and Cd and further showed that their interactions were additive. Similar findings were observed by Hambach et al. [60], revealing that co-exposure to Pb and Cd increases the relationship between cadmium and renal biomarkers. Exposure to heavy metals has been reported to negatively affect the function of the remaining functional nephrons [61]. These side effects could result in an elevation in cell death and glomerulosclerosis, which would constantly reduce the functional renal mass of the individual. As the urinary excretion of foreign compounds and toxicants reduces, the functional renal mass of the individual is reduced affecting the overall health of the individual [61].

### 4.6. Neurotoxicity

Neurotoxicity refers to any deleterious effect on the structure or function of the nervous system (central and/or peripheral) produced by an agent whether physical, chemical, or biological that reduces the potentiality of an organism to live or acclimatize to its environment [62]. The effects of acute exposure to neurotoxicants may be compensated by the nervous system, but a chronic exposure even to the least concentration may result in delayed brain damage [63]. These effects can, therefore, be seen in later stages of life even when the events that led to them occurred decades earlier. This implies that chronic exposure to low concentrations can possibly give rise to a nation with a lifetime loss of intelligence and motor capacities and permanent psychological disturbances [64]. According to Landrigan et al. [65], these effects can lead to a reduction in economic productivity, and when this is inherent in a nation, the resulting economic effects could be higher than the costs of controlling metal pollution. In the environment, mixtures of neurotoxic metals naturally occur, and metals exist in the environment as mixtures [8]. Pb, Cd, Hg, and As are thought to exhibit their neurotoxic effects [66,67] through common mechanisms, such as the production of reactive oxygen species (ROS) [68] and interaction with micronutrients [66,69,70].

Most metals are known to elevate the vulnerability to cognitive dysfunction and neuro-degenerative outcomes [71]. Cecil et al. [72] proved that the brain volume of children exposed to Pb was modified. Exposure to Pb during early postnatal life generates a higher discrepancy in learning performance than in older animals [73]. It has been evidenced that As induces hippocampal-dependent behavioral deficits in rodents [74] and report has shown that elevated levels of As alters growth and development in children resulting in neurological deficits [75]. Some Hg compounds have been shown to cause neurotoxicity, affecting the usual maturity of the central nervous system [76]. In vitro studies with animals have shown that methylmercury can affect the biological processes thought to be involved in Alzheimer’s disease [77]. Cadmium has also been established to generate free radicals in the brain [78]. Long-term occupational exposure to cadmium slows the psychomotor functions of the brain [79].

**Figure 6:**
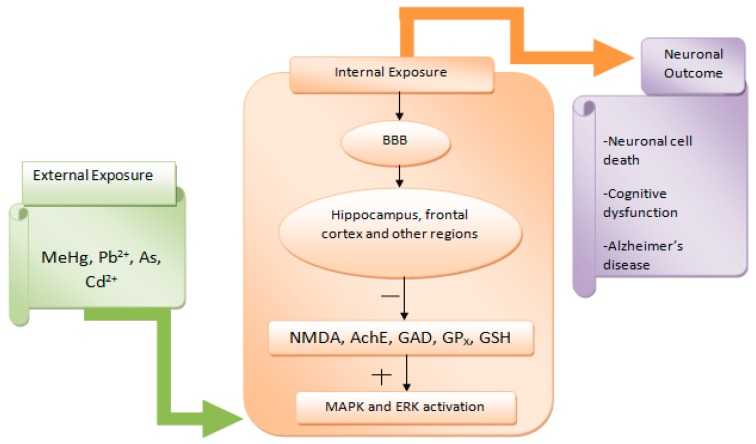
A conceptual framework of metal mixture exposure, mode of action, and disease outcome in the brain, the - sign indicates inhibition of cellular elements and the + sign rising apoptotic factors.

When metals are mixed, they show competitive interactions with macromolecule due to the similarity in their functions. The toxic interactions associated with metal mixture could be dose additive, interactive (synergistic or antagonistic), or sovereign to each other, which can produce elevated biochemical changes in several parts of the brain. It has been proven that sub-chronic exposure to a mixture of Pb, Cd, and As in albino rats caused neuronal developmental disorder by synergistic action [80]. The result revealed a major proof of the metal mixture’s neurotoxic activity and their possible relations. The possible relations of a metal mixture for passing the blood-brain barrier (BBB) gives internal exposure, critical for estimating the effective concentration of individual metal in the mixture responsible for potential risk of cognitive dysfunction [81]. Figure 6 shows the conceptual framework of exposure to a metal mixture, toxicology, and disease scenarios in the brain.

The effects of heavy metals may alter neurotransmission and cause neurodegeneration, which can show cognitive problems, disorders in movement, learning, and memory dysfunction. Epidemiological studies have shown that heavy metals induced neurotoxicity has been associated with several neurological diseases including Alzheimer’s disease (AD), amyotrophic lateral sclerosis (ALS), autism spectrum disorders (ASDs), gulf war syndrome (GWS), Huntington’s disease (HD), Parkinson’s disease (PD), multiple sclerosis, manganism, and Wilson’s disease [82,83,84,85,86,87]. While there is a scarcity of information on the combined effect of metal mixture exposure, few studies have established that neurotoxicity is an effect from exposure to Pb and other metals (Hg, As, Mn, and Cd) [88]. McDermott et al. [89] reported that prenatal exposure to Pb and As increases the chance of intellectual disability when combined with exposure to an individual metal. Co-exposure to high levels of Pb and Cd tends to affect mental and psychomotor development in children [88,90]. Co-exposure to Mn and Pb in the prenatal stage has been shown to distort the cognitive and language development in children at their second year compared with single metal exposure [68]. These studies have as well established that if symptoms are earlier recognized and adequate treatment and elimination from exposure is supplied, neurologic and or psychological function can remain stable or actually improve regardless of the initial exposure.

Metal mixture exposure occurs in several stages (embryo, fetus, newborn, child, adult, and old age), which is termed as windows of exposure by Karri et al. [91]. The level of internal dose of metal in a brain may have high inter-individual variability and high dependability on the anatomical and physiological development in the brain barrier system [92]. Additionally, exposure of Pb and Cd to pregnant rat has been reported to have an additive effect on decreasing sodium-potassium adenosine triphosphatase (Na+/K+ -ATP ase) function, in which Cd activity is potentiated by Pb for causing failure of the Na+/K+ -ATP ase pump. The Na+/K+ -ATP ase pump depletion forms the inhibition of intracellular K+, accumulation of intracellular Na+, and elevation in intracellular free Ca+2 leading to intensified cognitive dysfunction [93]. 

Karri et al. [91] established the common links between As, Pb, MeHg, and Cd to cause cognitive dysfunction. Pb2+ has been reported to obstruct with the glutamate (Glu) transmission and may distort the N-methyl-D-aspartate (NMDA) expression in the synaptic region. Studies with rats showed that arsenic affects the synaptic activity of neurons found in the hippocampus by hindering the NMDA function similar to Pb and upregulating the AchE function. MeHg inhibits the Glutamic acid decarboxylase (GAD) Glu transporter affecting the Glu uptake and NMDA over-expression [94]. MeHg also distorts the microtubules in the brain due to high affinity to binding the sulphur containing amino acids –SH [95].

### 4.7. Development of Cancer 

The World Health Organization in 2018 reported that cancer is the second leading cause of death in the universe today and was accountable for 8.8 million deaths in 2015. Globally, every one in six deaths results from cancer and approximately 70% of these deaths occur in low and middle-income countries. The frequency of individuals in many hospitals today with hormonal disorders could be as a result of heavy metal contamination in the environment, which has contributed to the disruption in the endocrine system [96].

The incidence and occurrence of cancer have been on the increase around the globe. In 2008, there were 12.7 million new cases and 7.6 million cancer-related deaths [97]. The newly reported cancer cases with 56% occurrence were in the developing countries and it is estimated that by 2030, 70% of all new cases of cancer will be found in developing countries [98]. Most of this increase in incidence is a consequence of population growth and increased life expectancy [99]. This risk may also result from overexposure to different heavy metals in the environment and their bioaccumulation for a long period of time, which is due to the increase in industrialization and urbanization in the developing countries. This effect can only be reduced when individuals start adopting a healthy way of life, when there is control in the occupational hazards and when there is a reduction in the exposure to heavy metals.

**Figure 7:**
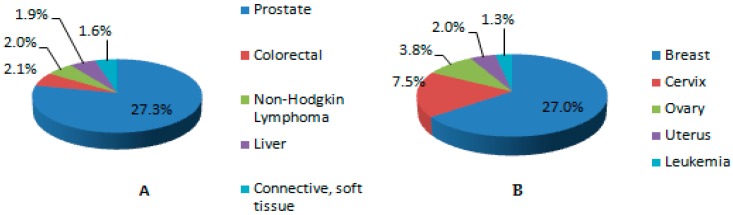
Five predominant cancers in both males (**A**) and females (**B**) living in Rivers State (Cancer in Nigeria, 2009–2013).

In Nigeria, the University of Port Harcourt Teaching Hospital (UPTH) Cancer registry, 2009-2013 reports the five most common cancers in both male and female living in Rivers State. The report shows that prostate cancer is the most occurring cancer in males, while breast cancer is the frequently occurring cancer in females. This is illustrated in Figure 7 below.

It has been estimated by Sylla and Wild [100] that one million Africans will die yearly from cancer-related causes by the year 2030. According to Sassman [101], cancer is the fourth leading cause of mortality and the eighth primary contributor to disability-adjusted life years (DALY) in South Africa. Despite the paucity of information on the cause-effect association between low dose metal mixture exposure and cancer, researchers have continued to find a relationship between the two.

Sassman [101] evaluated the dose-response connection between As and bladder cancer and reported an increased death from bladder cancer in areas exposed to As in their drinking water. Hopenhayn-Rich et al. [102] investigated the possible relationship between chronic environmental exposure to Pb and Cd and cancer incidence and reported higher incidences of gastrointestinal and lung cancers among individuals exposed to polluted rivers over 30 years. Wang et al. [103] established a strong relationship between pancreatic cancer and Cr, Se, and Mo by comparing the heavy metal composition of pancreatic juice collected from patients with pancreatic cancer exposed occupationally with others that were not exposed occupationally.

## 5. Public Health Effects of Heavy Metal Exposure in Sub Saharan Africa

The prevalence of mineral resources in SSA has resulted in threats in relation to environmental safety. Products of artisanal mining including heavy metals have caused environmental pollution as a result of poor regulation. In Zamfara State, Nigeria, an epidemic of lead poisoning from artisanal mining led to the deaths of about 163 people between March and June 2010, including 111 children under five years of age [104]. 

TerraGraphics Environmental Engineering (TG), World Health Organization (WHO), and Centers for Disease Control and Prevention (CDC), reported that approximately 400 children <five years old have been killed from the outbreak and thousands of people affected, including >2000 children left with permanent disabilities [105,106,107]. Dooyema et al. [107] measured the concentrations of soil Pb and soil Hg which showed >100,000 ppm and about 4600 ppm for Pb and Hg, respectively. The study found that surviving children < five years of age had blood Pb levels (BLL) of about 370 ug/dL which is above the CDC recommended BLL of 5 ug/dL [108].

While most studies in relation to the Zamfara Pb outbreak centred on clinical intervention and reduction of blood Pb level (BLL) in children, a study by Lo et al. [105] drew attention to some drawbacks like the non-assessment of Pb poisoning in livestock and other foods including dairy products. Since the populace may be exposed through a secondary pathway (i.e., through consumption of leaded foods), Lo et al. [105] suggested the importance of characterizing the magnitude of Pb distribution in livestock. Additionally, it is a known fact that lead does not exist as a single metal in the earth crust but occur as a combination with other heavy metal mixture on most cases of lead intoxication. These limitations formed the bedrock towards a research carried out Orisakwe [109] in Dareta and Abare, Zamfara State, Northern Nigeria.

The importance of various toxicant exposures is critical in global public health. Orisakwe et al. [109] reported that Pb may not be the only toxic metal of concern in the contaminated mining communities of Dareta, Abare, and Gasau of Zamfara State given the high levels of cadmium in meats and vegetables from these villages. The public health effects of exposure to lead either through ingestion or inhalation can cause damage to the brain, kidneys, bone marrow, and other body systems in young children. Blood Pb levels (BLLs) below 5 ug/dL have been shown to cause developmental problems including impaired cognitive function, behavioral difficulties, impaired hearing, and reduced stature in infants and children, while BLLs above 75 ug/dL has been implicated to cause coma, convulsions, and death [110]. Cd is an endocrine disruptor that crosses the placental barrier and accumulates in the foetus leading to neurodevelopmental toxicity [111].

Extraction of petroleum is one of the main causes of pollution in West Africa. Chindah et al. [112] and Oloruntegbe et al. [113] have reported the factors that result in the discharge of crude oil into the environment and they include oil pipeline corrosion, effluents from oil and gas industries and the recurrent act of damage to oil facilities in the South-South region of Nigeria resulting in contamination by heavy metals such as Pb, Zn, Cu, Cr, V, and Cd [113]. In a study assessing the link between industrial activities and pollution, Adekola and Eletta [114] attributed the high levels of Fe, Zn, Cu, Cr, and Mn in Asa River sediments in Nigeria to bottling, tannery, detergent, and other industries that discharge effluents into the river. In Ghana, contamination of water in the Iture estuary with Pb and Cd has been ascribed to waste carried by the Sorowie and Kakum River, which flow through a swiftly urbanized and industrialized central region [115].

Farming of food crops and vegetables in contaminated environments is also common in West Africa. This is done by small-scale farmers to maximize yields due to the seemingly high organic contents of waste dumpsite soils. Based on environmental studies over the past decade, it is clear that there is a steady accumulation of heavy metals in the African environment. The levels of pollution in many African countries are at dangerous points, as the present levels of many metals in water, soils, fish, vegetables, and food animals are above international limits [116].

Studies have recorded several effects of heavy metals in drinking water [117,118]. Smith et al. [119] detailed that drinking one liter per day water with As of 50 ug/L over an individual’s lifespan can possibly result to cancer of the liver, lung, kidney, or bladder in 13 per 1000 persons. Ahsan et al. [120] reported an improved incidence of skin lesions from As dose of 0.0012 mg/kg/day through drinking water. As has been reported to have a side effect on the central nervous system and cognitive development in children [121]. The central nervous system, renal, reproductive, neurological, cardiovascular, musculoskeletal, developmental and immunological systems have been reported to be affected by Pb [118]. Drinking water contaminated with Cd may lead to chronic renal failure [118,122]. Long-term exposure to low concentrations of Cd can possibly lead to deposition in the kidney, causing kidney disease, fragile bones, and lung damage [123]. 

Cancer risks have also been evaluated in Ghanian residents who eat foods produced from mining communities with soil showing increased levels of heavy metals [124]. Breast cancer risk has also been evaluated in a Nigerian population with volumes of tumors and body levels of Pb and other heavy metals [125]. The study also established evidence for interactions between Pb and Se. High Pb levels were directly proportional to tumor volumes in agreement with the identified tumour-inducing Pb effects, and selenium levels were inversely proportional to tumor volume, which is in conformity with its anti-proliferative effects. Hnizdo and Sluis-Cremern [126] reported the relationship between lung cancer and gold mining dust exposure to miners in South Africa. In a similar way in Southern Africa, McGlashen et al. [127] reported the correlation between lung, liver, oesophagal, and lymphatic system cancer with exposure to mining dust. According to Hnidzo et al. [128], individuals exposed to high mining dust stand a greater chance of having lung cancer. Similarly, in a Zimbabwean study, persons exposed to nickel during mining were found to be more vulnerable to having lung cancer risk [129].

Pb, Cd, Hg, and As are termed endocrine disrupting compounds [130] and exposure to them during pregnancy may have deleterious effects on the mother and unborn child [131]. It has been recorded by various studies that some adverse effects [132,133,134]. These heavy metals have been shown to influence the delicate maternal-fetal balance, hence causing long-term damage to the newborns [132,134].

Ajayi et al. [135] conducted an epidemiological research using 69 pregnant women including those who had previous spontaneous abortion history and control group without a record of recurrent spontaneous abortion. Blood samples were analyzed for heavy metals and results found indicated high levels of serum metals (Cd, Cr and Pb), which could cause recurrent spontaneous abortion. Additionally, Otebhi and Osadolor [136] reported a considerable increase in the blood toxic metals (Pb, Hg, Cd, and As) levels in pregnant women with a history of pregnancy complications compared with women who are also pregnant but without any record of pregnancy complications. Their findings were in conformity with other studies [135,137] where similar reports were closely related with spontaneous abortion. These findings show that increased serum heavy metals (Cd and Pb) can possibly lead to recurrent spontaneous abortion.

While there is a scarcity of literature on cases of heavy metal exposure in Nigeria, Orisakwe et al. [138] reported high concentrations of Pb, Cd, and Ni in some selected Nigerian fruits and vegetables. Results from this study concluded that from foods alone, the burden of Pb in an average Nigerian exceeds the values obtained in America and Europe. In the same vein, results gathered from the analysis of heavy metal content of some herbal remedies sold in Nigeria by Amadi et al. [139] showed high concentrations of Hg, Sn, and Sb in the products. These herbal remedies have been implicated to cause liver damage with a high incidence of mortalities and morbidities as reviewed by Amadi and Orisakwe [140].

Ideriah et al. [141] conducted research on the distribution of heavy metals in water and sediment along Abonnema shoreline, Nigeria. Their results showed that the shoreline was heavily contaminated as the concentrations of Cr, Zn, and Cu exceeded permissible limits set by the World Health Organization and therefore pose a serious health concern. Similarly, Owamah [142] assessed the heavy metals in a petroleum impacted river in the Niger Delta Region of Nigeria and reported that the levels of heavy metals, Cd, Cr, Cu, Fe, Ni, and Pb in River Ijana were generally above W.H.O. standards recommended for surface waters and concluded that this is an indication of pollution. Heavy metals discharged into the aquatic ecosystem are possible to be scavenged by particles leading to their buildup in sediments [143]. 

Some metals in trace amounts are biologically beneficial to the body such as Zn and Cu, while toxic metals build up in large quantities and cause deleterious health effects. Heavy metals occur as mixtures in the environment and may enter the body simultaneously through the air, water, or food. Once found in the human body, they accumulate rapidly and bio-accumulate leading to a rise in their concentration because they are not easily metabolized or excreted [144]. 

Nigeria crude oils have been studied by Akporido and Onianwa [144] and were reported to contain relatively appreciable concentrations of some heavy metals including Pb, Hg, Cu, Fe, Zn, and V. In Niger Delta, particularly in Port Harcourt, the arbitrary release of effluents by the petroleum companies into the environment constitutes a major factor to the degradation of the water and land ecosystem within its environs and contributes to the rise of the levels of heavy metals in this environment [145,146]. Toxicity arising from oil pollution can lead to many adverse effects in humans including respiratory illness, neurological, and kidney diseases [147]. Several studies have shown that heavy metal pollution has become a major characteristic trend in sub-Saharan Africa. 

Table 1 describes the heavy metal pollution from different sources such as soils, sediments, fish, vegetables, and water from various regions in sub-Saharan Africa, showing their associated public health effects including but not limited to brain damage, nephrotoxicity, hepatotoxicity, bone diseases, carcinogenicity, and others. Heavy metals such as Zn, Fe, Cr, and Cu are beneficial to the body, while Pb, Cd, Hg, and As have no known beneficial roles in the body [148].

In Niger Delta, Nigeria, Oze et al. [149] showed that high levels of heavy metals such as Pb and Cr in fish were above the WHO/UNEP/FAO standards of 0.29 ppm and 0.1 ppm, respectively, while Cd was found to be below the standard of 0.05 ppm. Farombi et al. [150] reported high metal concentrations of 3.4 ppm of Pb, 2.1 ppm of Cd, 5.0 ppm of Cu, 20.35 ppm of Zn, and 2.3 ppm of As. These concentrations were far above the WHO/UNEP/FAO limits of 0.29 ppm, 0.05 ppm, 0.5 ppm, 5.0 ppm, and 0.01 ppm for Pb, Cd, Cu, Zn, and As in fish.

Many heavy metals are naturally occurring elements in the environment and affect almost all the organs and tissues of the human system. Pb causes nephrotoxicity and neurotoxicity and also affects heme synthesis [151]. Cd can distort calcium metabolism, renal tubular dysfunction, bone diseases, and also lung cancer [152]. Mercury has deleterious effects on the immune and digestive systems. It also causes neurotoxicity [153]. 

Studies have shown that heavy metal mixtures may have joint effects that are significantly different from their individual effects [154,155]. Many studies have revealed the individual toxicity of these metals [156,157,158], but only a few studies have shown the mixture effects, which actually represent the real-life situation in the world [159,160,161]. In the need to mimic the real-life situation using multiple heavy metal exposure, Kentson et al. [159] observed the effects of one dose of heavy metal mixture oral administration on rats after four weeks of exposure. Their study showed that exposure to heavy metal mixtures induced toxic effects in the form of loss of body weight, disturbance in the hepatic injury and renal insufficiency, haematological system, abnormal neurological disorders, and animal death [159].

A study by Whittaker et al. [162] revealed that lowest observed effects levels of Pb, Cd, and As mixtures resulted in the improved incidence of mediators of oxidative stress such as delta-aminolevulinic acid (ALA), Cu, and Fe. Studies have established that the toxicity arising from exposure to metal mixtures on various organs and tissues in the body system including cytogenicity [17,163,164], oxidative stress [27], neurotoxicity [165,166], bladder cancer [167], toxicity on embryogenesis [168], immunotoxicity [27], and mortality [169].

High concentrations of heavy metals such as Pb, Cd, Cr, Zn, and Cu in vegetables have been reported by several studies in SSA. In Ethiopia, [170] reported 0.345 mg/kg of Cd, 130 mg/kg of Cu, 130 mg/kg of Zn and 24.11 mg/kg of Cr in vegetables. In Kano Nigeria, Abdullahi et al. [171] reported 13.19 mg/kg of Pb, 0.735 mg/kg of Cd, and 12.89 mg/kg of Cr in vegetables. A Zimbabwean study by [172] reported 6.77 mg/kg of Pb, 3.68 mg/kg of Cd, 0.05 mg/kg of Hg, 111 mg/kg of Cu, 221 mg/kg of Zn and 16.1 mg/kg of Cr in vegetables, while a Ugandan study by [173] recorded 18.7 mg/kg of Pb and 1.87 mg/kg of Cd. The concentrations of these heavy metals reported were higher than the WHO/FAO recommended limits for Pb 0.3 mg/kg; Cd 0.2 mg/kg; Cu 40 mg/kg; Zn 99.40 mg/kg; and Cr 1.30 mg/kg.

Similarly, high concentrations of heavy metals have also been recorded in the agricultural soils of many countries in SSA. In Kumasi, Ghana, Odai et al. [116] reported 54.6 mg/kg, 2.87 mg/kg, 2606 mg/kg, and 2606 mg/kg of Pb, Cd, Cu, and Zn, respectively, in soil. According to UNEP [174], a Kenyan study reported 264 mg/kg of Pb, 40 mg/kg of Cd, 18.6 mg/kg of Hg, 105 mg/kg of Cu, 462 mg/kg of Zn, and 157 mg/kg of Cr in soil. Fakayode and Olu-Owolabi [175] reported 92.07 mg/kg of Pb, 3.6 mg/kg of Cd, 37.9 mg/kg of Cu, 71.9 mg/kg of Zn, and 17.3 mg/kg of Ni in a Nigerian soil. Most of the heavy metals found in these agricultural soils were found to be higher than the WHO/FAO/EU permissible limits of Pb 10–70 mg/kg; Cd 0.07–1.1 mg/kg; Cu 6–60 mg/kg; Zn 50–100 mg/kg; and Cr 65 mg/kg for agricultural soils.

Several studies have also highlighted high concentrations of heavy metals in SSA waters. In Ghana, Fianko et al. [115] reported 0.075 mg/L of Pb, 0.041 mg/L of Cd, 2.45 mg/L of Cu, and 2.45 mg/L of Zn in water. A Kenyan study by Mireji et al. [176] recorded 0.496 mg/L, 0.01 mg/L and 1.95 mg/L of Pb, Cd and Cr respectively. In Niger Delta, Nigeria, the water has been found to be contaminated with heavy metal concentrations of 0.025–0.064 mg/L of Pb, 0.01–0.11 mg/L of Cd, 0.03–0.081 mg/L of Cr, and 0.03–0.09 mg/L of Ni [177]. A Zimbabwean study by [178] reported 1.02 mg/L, 0.12 mg/L, 2.48 mg/L and 2.37 mg/L of Pb, Cd, Cr, and Ni, respectively, in water, while Fatoki and Mathabatha [179] recorded 16.3 mg/L of Pb, 72 mg/L of Cd, 42.6 mg/L of Cu, and 27.6 mg/L of Zn in South Africa. These concentrations were higher when compared with the WHO permissible limits of 0.01 mg/L Pb, 0.003 mg/L Cd, 2.0 mg/L Cu, 3.0 mg/L Zn, and 0.07 mg/L of Ni for drinking water.

There are several factors that could attribute to the high concentrations of heavy metals found in agricultural soils, water, vegetables, and fishes around SSA which include traffic emissions, biomass burning and domestic combustion, waste disposal, illegal refining, and artisanal mining. Waste disposal is also a contributor to the high concentrations of heavy metals in SSA [180]. The open burning of waste at both the residential level and at dumpsites have been reported to release harmful air pollutants including heavy metals, dioxins, and polyaromatic hydrocarbons [181].

Heavy metal exposure may contribute to metabolic syndrome; though available data seem to be conflicting [182] because epidemiological data are largely cross-sectional; and variation in the study design, including samples used for heavy metal measurements, the age of individuals at which metabolic syndrome effects are measured. Metabolic syndrome defines the co-occurrence of factors that increase one’s risk for heart disease and other disorders such as diabetes and stroke 182]. A review by Planchart et al. [182] suggested that future studies, standardization, or increased consistency across study designs and reporting, and molecular mechanisms informed by model system studies are important to better evaluate potential causal links between heavy metal exposure and metabolic syndrome.

Pb is known to cause toxicity by replacing Zn in heme synthesis and depleting the role of heme synthesizing enzymes [183]. Individuals highly intoxicated by lead have been reported to show different forms of neurological syndrome including Pb palsy and encephalopathy, especially in children [184]. As leads to coagulation of proteins, the formation of complexes with coenzymes and inhibits the production of adenosine triphosphate (ATP) during respiration [185]. It is a probable carcinogen and high-level exposure can cause death [186]. Exposure to Cr has been reported by [187] to cause adverse effects to the skin including ulcerations, dermatitis, and allergic skin reactions.

Cd and its compounds can interfere in calcium metabolism, renal tubular dysfunction, or osteoporosis [152]. The correlation between Cd exposure and certain cancers have been evidenced in some epidemiological studies [188]. Cd has been reported to cause neurodegenerative disorders, breast cancer, diabetes, and prostate cancer [189,190,191]. Nickel has been evidenced to cause allergic contact dermatitis, oral cancer, asthma, reproductive toxicity, and carcinogenesis [192,193,194].

Pb is a non-beneficial element multi-organ toxicant [216]. Pb exposure resulting from both environmental and occupational activities has been identified as among the public health problems affecting the globe [217]. Young children are at high risk to lead because of their propensity to pick up particles from the ground and put into their mouths, and due to high levels of absorption of ingested Pb compared to adults [110].

Studies have reported that both long-term and short-term exposures to moderate levels of Pb are followed with some deleterious effects [218,219]. Blood Pb levels ≤40 ug/dL of US occupational exposure limit guidelines have been implicated to cause both systolic and diastolic hypertension among women aged between 40–59 years old [220]. According to the National Research Council report [221] 1993, children, pregnant women and breastfeeding mothers were grouped to be more susceptible to Pb exposure due to high bone turnover associated with these physiological states. It has been evidenced by some researchers that bone Pb stores add to the circulating levels of Pb in blood particularly, in pregnant women [222,223]. This is as a result of the mobilization of Pb stored in the bones, especially, in individuals with low Ca intake [222]. Studies have reported that maternal BLLs ≤ 10 ug/dL may result in problems during pregnancy, such as increased risk of high blood pressure, miscarriage, reduced length of gestation, spontaneous abortion, and premature delivery [224,225].

Children living in SSA have an obvious risk for Pb poisoning [226]. Table 2 describes the blood lead levels (BLLs) and public health effects in SSA. Njoku and Orisakwe [11] reported that 78.9% of expectant mothers in Nigeria had BLLs ≥10 ug/dL (range: 0.5–448 ug/dL), while Adekunle et al. [227] and Ugwuja et al. [228] reported that the geometric mean of Nigerian pregnant women (15–40 years old) ranges between 2.7 and 73.8 ug/dL. Pb levels in cord blood varied from 2–17 ug/dL in South Africa [229]. The values obtained from these countries in SSA are higher than those reported in North Carolina women (USA) (0.07–0.13 ug/dL) [230] or from the Duke cohort (>75% of pregnant women with BLLs <1.00 ug/dL) [231] or from NHANES 2003–2004 (1.78 ug/dL) or in Quebec (1.50 ug/dL) [230]. It is seen from these facts that young children resident in SSA are more probably exposed to lead in utero than US children. The high mean BLLs shown in SSA children may be indicative of elevated levels of Pb in their environments [231].

Despite the report by Adeniyi and Anetor [232] that the general Nigerian population has high blood levels, there is still a scarcity of data on the BLLs in Nigerian pregnant women. The data from Table 2 shows that the BLLs of the Nigerian population including children and pregnant women are high. The high incidence of increased blood Pb among children and pregnant women may be indicative of high Pb content in Nigeria’s gasoline [233], which was estimated at 0.66 g/L [175]. It could also be attributed to increased use of petrol-powered generating set, causing lead pollution [228]. Furthermore, the high incidence of elevated BLLs in Nigerian pregnant women could result from the consumption of contaminated water and foods, inhalation of poor indoor air, and unregulated use of cosmetics [234].

## 6. Risk Assessment of Exposure to Mixtures of Heavy Metal

The mechanism of action of the metal is important while assessing the risk of heavy metal mixtures [244,245]. The mechanism of action refers to the methods by the interaction of the toxicant with the receptor and the progress through changes in the organism that leads to sub-lethal and deadly effects. According to Borgert et al. [246], it is the response shown by an organism exposed to a pollutant or the key features of the mechanism needed for the production of a biological response. The means of action is needed in the estimation of the toxicity of mixtures of toxicants in the assessment of risk. 

The difference in the concentration of single metals in mixtures is the underlying factor causing the non-feasibility in assessing every metal mixture combination [247]. Many models have been used to mimic the toxicity of metal mixtures to organisms. Most of these models are dependent on the concepts of independent action (IA) and concentration additions (CAs) [248]. These models are based on several theories linked to the modes of action of a compound. The CA is used when two or more chemicals have a connected mode of action, for example, when they aim at the same enzyme. CA is based on the theory of dilution and assumes that any constituent of a mixture can be replaced by an effective concentration of another constituent, without changing the total effect of the mixture [249]. Independent action (IA) is used when two or more chemicals have dissimilar modes of action [250]. It is based on the principle of independent random events. It is assumed that the susceptibilities of an organism to each of the chemicals in the mixture are statistically independent.

Both CA and IA depart from the idea that substances do not interact at target sites. However, this prediction is not always satisfied because substances can increase or decrease each other’s toxicity, i.e., substances may interact when combined in a mixture. If the observed mixture effect is more or less than additive than the expected based on the reference model, the mixture acts either synergistically or antagonistically respectively [251,252]. 

Studies on metal mixture toxicity have revealed that mixture effects are hard to predict as all potential outcomes have been observed [253,254]. Studies have revealed that interactions can be conflicting across various experiments [253], or that interactions can be dependent on concentration [255,256].

Mixture effects and mixture interactions from chronic tests vary from those of acute tests because acute tests do not account for metal interactions taking place during longer-term detoxification. Some studies have concluded that there is a paucity of information on the validity of the mixture reference models for chronic metal mixture toxicity at low concentration doses to permit the addition of metal mixture toxicity in risk assessment frameworks [257,258].

A study by Spurgeon et al. [259] has explained the mechanisms underlying chemical mixtures by proposing a biologically based framework that shows the idea of external exposure. This idea explains the interaction of mixtures in the environment, its exposure and uptake by the host organism (toxicokinetic), to the expression of toxicity in the host organism (toxicodynamics), and finally, to the combined toxic effect known as toxicogenomics. Toxicogenomics is a recent scientific field, which shows how genomes respond to environmental pollutants. It explains the molecular mechanisms looking at both toxicity and biomarkers that reveal genetic vulnerability to toxicants [260]. Toxicogenomics is important because environmental pollutants such as heavy metals contain more than one mechanism of action and may interact with more than one specific site along an adverse outcome pathway [261]. The adverse outcome contains aspects of molecular interactions, followed by issues of responses to stress due to exposure to the toxicant, and finally, to deleterious effects resulting from exposure to the joint mixture [3].

## 7. Conclusions

It is clear from this review that evaluating exposures on an individual chemical basis does not adequately account for the wide array of mixtures encountered in the environment [262,263]. While there is paucity of information on epidemiological evidence to heavy metal mixture exposures and associated health effects in SSA, it is non-indicative of the increased level of heavy metal pollution in SSA due to several manmade activities in the region which include artisanal mining, illegal refining, and others. The prevalence of mineral resources in SSA has resulted in threats in relation to environmental safety. Products of artisanal mining including heavy metals have caused environmental pollution as a result of poor regulation. This is because health regulations concerning these exploratory activities are inadequate and feebly enforced. Additionally, urbanization and industrialization contribute largely to heavy metal pollution as seen from the reviewed literature above. With significant evidence from both occupational and environmental exposure to metal mixtures, it is imperative to note that low dose metal mixtures can cause deleterious effects to man.

In order to reduce the environmental and public health effects of heavy metal pollution, government and health agencies need to give additional attention to the environment and anthropogenic activities. There is need for regulatory authorities in SSA to be stricter in enhancing and enforcing existing rules in order to protect humans from heavy metal exposure resulting from the environment. Furthermore, since heavy metals occur as heterogeneous mixtures in the environment and are ubiquitous, there is need for researchers to start looking for an alternative medicare since heavy metals have been shown to ward off treatment with modern medicine due to their toxicity.

## Figures and Tables

**Table 1:** Heavy metal pollution in Sub-Saharan Africa.

Country/Region	Pollution Source	Pb	Cd	Mn	Cu	Fe	Zn	Cr	Ni	As	Ref.
Nigeria/Kaduna	Soil	0.87–1.41	0.0014–8.02		13.21–42.15	1425.0–1981.6	10.10–112.04	21.35–358.00			[166]
Nigeria/Kaduna	Vegetable	0.0014–0.001	0.013–2.12		0.582–8.65	331.6–1252	14.19–69.07	0.058–2.80			[166]
Nigeria/Niger Delta	Water	39 ± 33	2.4 ± 3.1		16.1 ± 7.4		107 ± 7.9	42 ± 17			[144]
Nigeria/Ibadan	Water	0.162–0.195	0.279–0.315		8.744–10.307		5.063–5.096	0.052–0.059	0.103–0.133		[195]
Nigeria/Benin	Soil	227 ± 160	2.0 ± 2.9				562 ± 510	94.5 ± 150			[196]
Nigeria/Akwa Ibom	Fish	0.013 ± 0.003	0.011 ± 0.004		81.36 ± 5.06		223.0 ± 23.47	0.044 ± 0.05		0.017 ± 0.02	[197]
South Africa/Pretoria	Soil	12.8–145	0.09–0.98		33.9–140	39.3–97.6	43.6–101				[198]
Kenya/Nairobi	Vegetable	0–2.4	0–3.02		0.52–21.34		20.13–89.85	0–1.24			[199]
Kenya/Nairobi	Soil	0.57–20	0–2.6		3.59–75.37		14.62–198.3	0.03–1.4			[199]
Ghana/Accra	Soil	184.44	103.66		202.99				72.00		[200]
South Africa/Philippi horticultural area	Water	0.04 ± 0.006	0.01 ± 0.002	0.02 ± 0.002	0.02 ± 0.003		0.02 ± 0.003	0.06 ± 0.009	0.02 ± 0.002		[201]
South Africa/Philippi horticultural area	Soil	19.24 ± 2.91	0.74 ± 0.18	96.74 ± 12.29	14.53 ± 2.02			30.13 ± 3.93	1.71 ± 0.40		[201]
South Africa/Philippi horticultural area	Vegetable	2.32 ± 0.91	0.22 ± 0.09	41.64 ± 5.21	5.55 ± 0.57		54.12 ± 9.24	2.68 ± 0.52	0.34 ± 0.25		[202]
Zimbabwe/Bulawayo	Water	0.19 ± 0.03	0.06 ± 0.03								[202]
Zimbabwe/Bulawayo	Sediment	51.67 ± 2.36	7.33 ± 0.76		79.17 ± 7.64			108.33 ± 17.02			[202]
Zimbabwe/Bulawayo	Fish	35	5		120			10			[202]
Cameroon/Yaounde	River sediment	20.3–249	2.8–15.6		42.8–142		26.8–341	94.7–199	2.68–32.7		[203]
Ethiopia	Vegetable		0.345		130		130	24.11			[170]
Ghana	Fish		0.028							2.31	[204]
Ghana/Iture	Water	0.075	0.041		2.45		2.45				[115]
Ghana/Kumasi	Soil	54.6	2.87		2606		2606				[116]
Ghana/Tarkwa	Water									1.3	[205]
Kenya	Water	0.496	0.01					1.95			[176]
Kenya/Nairobi	Soil	264	40		105		462	157			[174]
Namibia	Sediment				10500		205		1950		[206]
Nigeria	Herbal medicines	27	4.75			97.5	25.5		78		[207]
Nigeria/Calabar	River sediment	20	0.2		64	15	184	65	67		[208]
Nigeria/Ibadan	River surface water	0.046	0.0044		0.0033	0.018	0.14		0.0031		[209]
Nigeria/Kano	Vegetable	13.19	0.735					12.89			[171]
Nigeria/Lagos	Soil	67.5–426	1.61–5.31								[210]
Nigeria/Niger Delta	Water	0.025–0.064	0.01–0.11					0.03–0.081	0.03–0.09		[177]
Nigeria/Niger Delta	Fish	0.3	0.03					0.53	0.21		[149]
Nigeria/Ogun	Fish	3.4	2.1		5		20.35			2.3	[150]
Nigeria/Osogbo	Soil	92.07	3.6		37.9		71.9		17.3		[175]
South Africa	Water	16.3	72		42.6		27.6				[175]
Tanzania	Vegetable	4.9	0.3								[211]
Tanzania, along Lake Victoria	Water sediment	54.6	7		26.1		83.7	12.9			[212]
Uganda	Vegetable	18.7	1.87								[173]
Uganda, along Lake Victoria	Water	1.44	0.02			0.16		0.02	0.13		[213]
Zambia	Sediment				12,855 ± 1445	1030 ± 58					[214]
Zambia	Sediment	9.75	0.8				125	130	220		[215]
Zimbabwe	Water	1.02	0.12					2.48	2.37		[178]
Zimbabwe Harare	Vegetable	6.77	3.68	0.05	111		221	16.1			[172]

**Table 2:** Blood lead levels (ug/dL) and public health effects in Sub-Saharan Africa.

S/N	Age Groups	Sex	Place of Study (City/Country)	Condition	Reported Concentrations	Disease Scenarios	Exposure Scenarios	Ref.
1	Adults	F	Abeokuta, Southwest Nigeria	Pregnant	54.50 ± 4.4	Spontaneous abortion, premature delivery, pregnancy complications, still birth, hypertension, low birth weight	Non-occupationally exposed	[234]
2	Adults	M	Nkpor, Nigeria	N/A	39.00 ± 4.00	Increases risk of hypertension and liver damage	Occupationally exposed	[235]
3	Adults	M	Nkpor, Nigeria	N/A	17.00 ± 4.00	Increases risk of hypertension and liver damage	Non-occupationally exposed	[235]
4	Adults	F	Niger Delta, Nigeria	Pregnant	40.00 ± 16.50	Spontaneous abortion, premature delivery, pregnancy complications, still birth, hypertension, low birth weight	Non-occupationally exposed	[228]
5	Adults	F	Niger Delta, Nigeria	Non-pregnant	27.7 ± 1.10	Hypertension, increased risk of renal failure, cardiovascular attacks	Non-occupationally exposed	[236]
6	Adults	M/F	Oshodi, Dopemu, & Ojota in Southwest Nigeria	N/A	155.42 148.56 122.6	Increases risk of hypertension and liver damage, endocrine disorder, reproductive disorder	Occupationally exposed	[237]
7	Adults	M	Port Harcourt, Nigeria	N/A	50.37 ± 24.58	Increases risk of hypertension and liver damage	Occupationally exposed	[238]
8	Adults	M	Port Harcourt, Nigeria	N/A	41.40 ± 26.85	Increases risk of hypertension and liver damage	Non-exposed	[238]
9	Children	M/F	Nigeria	N/A	>10 or >20	Increases risk of hypertension and liver damage	Non-exposed	[239]
10	Adults	M	Abeokuta, Nigeria	N/A	48.50 ± 9.08	Increases risk of hypertension and liver damage	Exposed	[240]
11	Adults	M	Southwest, Nigeria	N/A	63.00 ± 9.00	Increases risk of hypertension and liver damage	Exposed	[241]
12	Adults	M	Southwest, Nigeria	N/A	61.00 ± 11.00	Increases risk of hypertension and liver damage	Non-exposed	[241]
13	Children	M/F	Allada, Benin Republic	N/A	46.6	Lowers intelligent quotient scores, aggressive and violent behaviours	Environmentally exposed	[242]
14	Adults	F	Allada, Benin Republic	Non-pregnant	55.1	Increases risk of hypertension and liver damage	Environmentally exposed	[242]
15	Adults	M	Kinshasa (Democratic Republic of Congo)	N/A	127	Increases risk of hypertension and liver damage	Environmentally exposed	[243]
16	Children	M/F	Kinshasa(Democratic Republic of Congo)	N/A	11.5	Lowers intelligent quotient scores, aggressive and violent behaviours	Environmentally exposed	[243]
17	Children	M/F	Northwest, Nigeria	N/A	143.8	Lowers intelligent quotient scores, aggressive and violent behaviours	Environmentally exposed	[107]
18	Adults	F	Abakaliki, Nigeria	Pregnant	40.0 ± 16.5	Spontaneous abortion, premature delivery, pregnancy complications	Environmentally exposed	[228]
